# Dissociable effect of different measures of Vascular Metrics on Cognitive Function: The Mediating role of the Thalamic Subregions

**DOI:** 10.1002/alz.092298

**Published:** 2025-01-09

**Authors:** Srijan Konwar, Martina Bocchetta, Matteo De Marco, Annalena Venneri

**Affiliations:** ^1^ Brunel University London, Uxbridge UK; ^2^ Dementia Research Centre, Department of Neurodegenerative Disease, UCL Queen Square Institute of Neurology, University College London, London UK; ^3^ Brunel University London, Uxbridge, UB8 3PH UK; ^4^ Brunel University London, London UK; ^5^ University of Parma, Parma Italy

## Abstract

**Background:**

The impact of white matter hyperintensities (WMH) on cognitive performance is uncertain due to inconsistent findings. The thalamus is of particular interest given its susceptibility to vascular damage. To test how different vascular risks (Deep/Periventricular WMH and Vascular Scores (VS)) affect thalamic subregional volumes and whether that has any mediating effect on different cognitive domains in healthy controls, mild cognitive impairment (MCI) and AD.

**Method:**

A sub‐sample of 93 participants (age = 64.96 ± 10.847, sex = 49 males/44 females) included controls (n = 40), MCI (n = 28) and AD (n = 25) were selected from the larger multi‐site VPH‐DARE cohort. 3T1‐weighted MRI images were pre‐processed using Freesurfer and its module to segment into 6 thalamic regions and its subcomponents. MD values of the bilateral posterior thalamic radiation (PTR) were extracted. Lexical semantic, episodic and executive functions were assessed. A non‐parametric mediation analysis was run using a bias‐corrected percentile bootstrapped with 5000 permutations controlling for covariates. A complementary analysis was conducted using the mean diffusivity (MD) values of the left and right PTR as a mediator.

**Result:**

A indirect effect of VS on the midline regions led to poor performance across all three cognitive domains: lexical (β = –0.077, p = 0.045), episodic (β = –0.097, p = 0.028) and executive (β = –0.079, p = 0.048). VS had a indirect effect on lexical (β = –0.097, p = 0.040) and episodic (β = –0.153, p = 0.005) tests through MD of the left PTR. Similarly, both deep (β = –0.249, p = 0.016) and periventricular (β = –0.309, p = 0.002) WMH resulted in a indirect effect on episodic memory. Only VS resulted in a negative indirect effect on episodic memory (β = –0.121, p = 0.020) through the MD of the right PTR.

**Conclusion:**

Higher VS resulted in poor cognitive performance by impacting subregional volumes of the thalamus and white matter integrity of tracts. The effect of both deep and periventricular WMH on cognitive performance appears to be mediated by the white matter integrity of the left thalamic tracts more than thalamic sub‐regional volumes possibly reflecting different vascular mechanisms.

